# Poor bone health in Duchenne muscular dystrophy: a multifactorial problem beyond corticosteroids and loss of ambulation

**DOI:** 10.3389/fendo.2024.1398050

**Published:** 2024-11-28

**Authors:** Amelia Hurley-Novatny, David Chang, Katsuhiro Murakami, Ling Wang, Hongshuai Li

**Affiliations:** ^1^ Department of Orthopedics and Rehabilitation, University of Iowa, Iowa City, IA, United States; ^2^ Department of Anatomy and Cell Biology, University of Iowa, Iowa City, IA, United States; ^3^ Medical Scientist Training Program, Carver College of Medicine, University of Iowa, Iowa City, IA, United States

**Keywords:** DMD, skeletal abnormality, osteoporosis, myokines, muscle and bone crosstalk

## Abstract

Duchenne muscular dystrophy (DMD) is a progressive, fatal muscle wasting disease caused by X-linked mutations in the dystrophin gene. Alongside the characteristic muscle weakness, patients face a myriad of skeletal complications, including osteoporosis/osteopenia, high susceptibility to vertebral and long bone fractures, fat embolism post-fracture, scoliosis, and growth retardation. Those skeletal abnormalities significantly compromise quality of life and are sometimes life-threatening. These issues were traditionally attributed to loss of ambulation and chronic corticosteroid use, but recent investigations have unveiled a more intricate etiology. Factors such as vitamin D deficiency, hormonal imbalances, systemic inflammation, myokine release from dystrophic muscle, and vascular dysfunction are emerging as significant contributors as well. This expanded understanding illuminates the multifaceted pathogenesis underlying skeletal issues in DMD. Present therapeutic options are limited and lack specificity. Advancements in understanding the pathophysiology of bone complications in DMD will offer promising avenues for novel treatment modalities. In this review, we summarize the current understanding of factors contributing to bone problems in DMD and delineate contemporary and prospective multidisciplinary therapeutic approaches.

## 1 Introduction

Duchenne Muscular Dystrophy (DMD) is a debilitating disease characterized by progressive muscle wasting due to X-linked recessive mutations in the dystrophin (*DMD*) gene (1). While the decline in motor function is well-documented, DMD patients face a myriad of other challenges, including intellectual disabilities, depression, delayed puberty, gastrointestinal complications, and skeletal abnormalities (2). Among these, skeletal issues such as osteoporosis, fragility fractures, scoliosis, short stature, and fracture-associated fat embolism (3) significantly impact patients’ quality of life (1, 2).

Poor skeletal health is one of the most overlooked aspects of this disease despite causing considerable morbidity. Low bone mineral density (BMD) (osteoporosis/osteopenia) is prevalent in DMD boys, especially those on glucocorticoid treatment, which poses a significant risk of pathological fractures (2, 4). Up to 60% of patients experience low-trauma extremity fractures (usually the distal femur, tibia, or fibula) by age 15 (5), and vertebral fractures occur in 30% to 60% patients (6). If left untreated, vertebral fractures can lead to chronic back pain and spine deformity, while leg fractures can cause premature, permanent loss of ambulation (2, 7, 8). Death due to fat embolism syndrome after long-bone fractures has also been reported in DMD boys (1, 3, 9). Moreover, reduced growth and short stature are also common (10, 11), further adding to the burden of skeletal issues in DMD.

**Figure 1:**
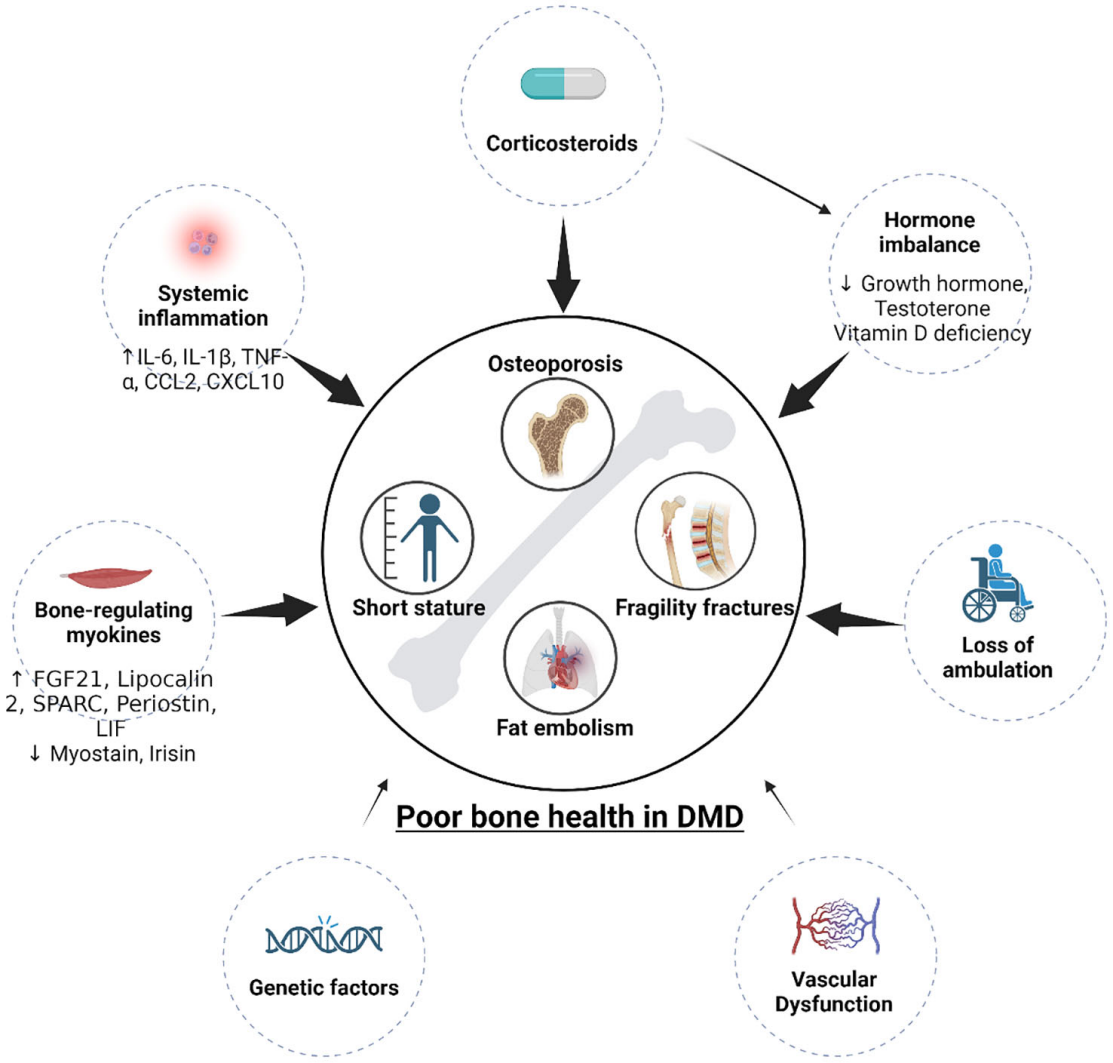
Pathogenesis (contributors) of poor bone health in DMD. Multiple factors contribute to short stature and osteoporosis. Corticosteroids, loss of ambulation, vascular dysfunction, hormone imbalance (GH and testosterone deficiencies), systemic inflammation, and dystrophic myokines all contribute to the poor bone health in DMD. Genetic factors that may contribute to poor bone health has not been explored.

The cause of poor bone health in DMD is complicated. It was previously thought that corticosteroid use and decreased mechanical stimulus from loss of ambulation (12), both of which are well-known to independently affect bone health (13), are the primary drivers of bone loss in DMD. These factors both substantially contribute to osteoporosis and increased fracture risk but do not completely explain the pathogenesis. Patients still develop osteoporosis and experience fractures without corticosteroid treatment and before loss of ambulation (12, 14). Thus, poor bone health cannot be explained solely by corticosteroid use or loss of ambulation, indicating that there are other mechanisms contributing to these aspects of disease. Indeed, recent evidence suggests that the pathogenesis of poor bone health is far beyond corticosteroid usage and muscle weakness, implicating nutritional deficiencies, hormonal imbalances, systemic inflammation, myokine release from dystrophic muscle, and vascular dysfunction in contributing to the deterioration of bone health to varying degrees, as summarized in **Figure 1** (15–18). These factors, to varying degrees, disturb bone homeostasis by preventing bone deposition by osteoblasts and promoting bone resorption by osteoclasts, thus disrupting BMD and bone microarchitecture in DMD patients (18–21). Further understanding of these mechanisms will help us better understand DMD’s impact on bone health, identify novel therapeutic targets, develop therapeutic approaches, and better manage this debilitating issue.

We currently lack comprehensive guidelines for managing bone problems in DMD. The DMD Care Considerations Working Group (DCCWG) recommends lateral spine radiographs to detect vertebral fractures every 2-3 years in corticosteroid-naïve patients and every 1-2 years for those treated with corticosteroids (22). However, monitoring of bone mineral density (BMD) via dual energy X-ray absorptiometry (DEXA) scanning is not recommended as routine management since there is no established correlation between BMD and fracture risk for this population (2, 23). Therapeutic intervention in the form of intravenous bisphosphonates is primarily recommended after detection of severe pathological fractures (2). Excitingly, emerging strategies, including physical therapy (PT), innovative bone-sparing corticosteroids, anti-resorptive agents, hormone replacement therapy, and targeted antibodies offer promise for improving bone health in DMD.

Despite the significant impact on patient’s lives, skeletal health in DMD remains an understudied area. In this review, we aim to critically analyze existing evidence, providing an up-to-date assessment of risk factors, pathogenic mechanisms, and emerging therapeutic avenues for addressing skeletal issues in DMD.

## 2 Risk factors and mechanisms of poor bone health in DMD

### 2.1 Immobilization and reduced mechanical loading

As DMD progresses, patients experience a gradual loss of mobility, often leading to wheelchair use by the second decade of life. Bone health is closely linked to mechanical stimulus (13), with increased loading promoting bone deposition while chronic unloading favors bone resorption (24). As DMD advances, there is diminished force on the skeleton. This is especially pronounced in the transition from the ambulatory to non-ambulatory phase, after which there is a marked decrease in BMD (12). This reduction in mechanical loading has long been considered a primary driver of impaired bone health in DMD patients.

### 2.2 Corticosteroid side effects

Corticosteroids, namely prednisone and deflazacort, are mainstay therapies in DMD, albeit non-curative. Their use has revolutionized DMD treatment and significantly improved survival rates by delaying the need for mechanical ventilation and preserving cardiac function (6, 25–27). They prevent muscle deterioration, which is thought to be by improving sarcolemma healing and reducing inflammation (27–29). However, their long-term use is associated with significant adverse effects, including osteoporosis, pan-hormonal suppression, mood disturbances, and metabolic dysfunction (6, 30–33). Numerous studies have demonstrated increased fracture risk and growth retardation in corticosteroid-treated patients (5, 25, 30, 32, 34–37), with earlier initiation and longer treatment durations correlating with higher risk (36, 38, 39).

Long-term corticosteroid use is known to cause osteoporosis through both direct and indirect mechanisms. Corticosteroids directly inhibit bone formation and increase bone resorption by acting on osteoblasts and osteoclasts (40, 41), leading to impaired bone microarchitecture and low BMD (19). On average, 6-12% of BMD is lost in the first year of corticosteroid treatment and 3% is lost every year after that (41). Beyond their direct effects on osteoblasts and osteoclasts, corticosteroids impair intestinal calcium absorption (42) and contribute to vitamin deficiency (4, 43), which indirectly affect bone homeostasis.

Corticosteroids also significantly contribute to growth retardation in DMD patients. Although growth retardation occurs in corticosteroid-naïve DMD patients through mechanisms which are not fully understood (10, 44), corticosteroid usage significantly worsens this manifestation of the disease (39, 45–47). Corticosteroids exert direct toxicity to the growth plate by inducing chondrocyte apoptosis (48, 49). They further affect growth plate physiology by interfering with the growth hormone (GH) – insulin-like growth factor-1 (IGF-1) axis. Corticosteroids inhibit endogenous GH production (42, 50) and antagonize the peripheral action of GH and IGF-1 (51). Furthermore, GH and testosterone act synergistically to promote bone growth during puberty (52). Corticosteroid-induced hypogonadotropic hypogonadism and consequent testosterone deficiency further impair the partnership of the GH-IGF-1 axis and testosterone, which substantially contributes to corticosteroid-induced short stature if used before puberty (47, 52).

### 2.3 Vitamin D deficiency

Studies have shown that up to 84% of DMD patients have a documented vitamin D deficiency at some point, even with supplementation (5, 38). Multiple aspects of disease may contribute to this phenomenon. Loss of ambulation is linked to lower vitamin D levels, possibly due to reduced sunlight exposure (14). Corticosteroids may be a major contributor to vitamin D deficiency and resistance to supplementation by increasing breakdown (4, 43). DMD patients also have elevated fat mass, which increases vitamin D distribution into fat and reduces its availability (53–55). Additionally, lower serum vitamin D binding protein levels were observed in DMD patients, which reduces vitamin D stores, further explaining resistance to supplementation (56, 57). Despite a high prevalence of vitamin D deficiency in DMD patients, the relative impact of vitamin D supplementation on bone health in this patient population is still elusive. Retrospective and cohort studies have not consistently correlated supplementation with improved BMD or reduced fracture risk (5, 16), questioning the importance of vitamin D deficiency as a contributor to poor bone health in DMD. However, resistance to supplementation demonstrates a need for more effective strategies to reach adequate vitamin D levels in this population.

### 2.4 Systemic inflammation

Muscle degeneration and systemic inflammation are characteristic features of DMD (58, 59), leading to increased circulating levels of pro-inflammatory cytokines, which in turn negatively impact bone metabolism (60). Elevated levels of inflammatory cytokines, including interleukin (IL)-1β, IL-6, tumor necrosis factor α (TNF-α), chemokine ligand 2 (CCL2), and CXC motif chemokine ligand 10 (CXCL10) have been reported in DMD animal models and patients, which may contribute to bone loss in DMD (summarized in **Table 1**) (59, 61, 62). IL-1β, IL-6, and TNF-α all have been shown to increase bone resorption by stimulating osteoclast activity and differentiation (63). Likewise, CCL2 and CXCL10 also affect osteoclastogenesis and increase bone resorption (64–66). So far, the effects of these cytokines on bone metabolism have been mostly studied in inflammatory, autoimmune and cancer conditions. Only IL-6 has been directly studied in DMD, showing increased osteoclast formation in *ex vivo* murine calvaria bone cultures treated with mdx mouse serum, which was reduced following treatment with IL-6 neutralization antibody (7).

**Table 1:** Cytokines and myokines involved in bone loss in DMD.

Cytokine/myokine	Effects on bone	Effects on muscle	Levels in DMD	Benefit of inhibition in DMD
IL-6	Increases osteoclast differentiation and osteoblast differentiation, but with a net resorptive effect leading to osteoporosis (63, 67, 102)	Muscle degeneration, inflammation, exhaustion of stem cells, fibrosis, adipogenesis (103)	Increase (7, 59, 62, 104, 105)	Mdx mice treated with anti-IL-6 had increased muscle diameter, decreased fibrosis, lower serum CK. No effects on diaphragm or heart (104). Calvaria explants treated with DMD patient serum had attenuated bone resorption when treated with anti-IL-6 (7)
TNF-α	Suppresses bone formation, induces resorption (102)	Frequently induces atrophy, but is involved in muscle regeneration in certain contexts (106)	Increase (59, 62, 105)	Inhibition reduces muscle damage and degeneration (107, 108) Reduces fibrosis but decreases ejection fraction and left ventricle thickness (109)
IL-1β	Suppresses bone formation, induces resorption (102)	Induces skeletal muscle catabolism (110)	Increase (59, 62, 105)	IL-1Ra inhibition in mdx mice improved forelimb grip strength but did not affect EDL function (111)
CCL2, CXCL10	Induces resorption (64–66)	CCL2 stimulates regeneration at low concentrations, but inhibits regeneration at too high of concentrations (112, 113) CXCL10 increases myogenic differentiation *in vitro* but has no effect *in vivo* (114)	Increase (61, 62, 105)	CCL2 inhibition reduced diaphragm inflammation (115) CXCL10 has not been studied.
RANKL	Elevated RANKL induces bone resorption (63, 116)	Implicated in pathogenesis of hypertrophic cardiomyopathy, inhibition improves muscle function in a variety of disease states (117)	Decrease (73, 118)	Improves cardiac function (119) Improves skeletal muscle function (72) Treats osteoporosis (72, 120, 121) in dystrophic mice and DMD patients
FGF21	Likely promotes resorption (76, 79, 122, 123)	Promotes muscle atrophy (75)	Increase (62, 77)	Inhibition improves osteoporosis (77)
Myostatin	Induces osteoporosis (67, 87, 89, 124)	Negatively regulates muscle fiber number and size (84)	Decrease (62)	Improved muscle strength, BMD in mdx mice (88, 125–128) Trend improvement in muscle and bone in humans, trial prematurely ended due to adverse effects (94, 129)
Irisin	Increases bone growth, BMD (96)	Promotes hypertrophy, prevents atrophy (97)	Decrease (62)	Supplementation improved muscle strength, mass in mdx mice (130)
Lipocalin 2	Inhibits linear growth, inhibits bone deposition, and induces bone resorption (101)	Inhibits myogenesis (131)	Increase (62, 100)	Increased trabecular volume, improved grip strength, reduced diaphragm fibrosis (100)
SPARC, periostin	Thought to be pro-osteogenic (132–134), but effect as a myokine has not been evaluated	SPARC: unclear, may be pro- or anti-regenerative, may be dependent on context, alters metabolism (135–137) Periostin may inhibit myogenesis, promote fibrosis (138, 139)	Increase (62)	Has not been studied
Other elevated myokines – LIF, OSM	Overexpression leads to osteopetrosis, knockout leads to smaller bones (140)	Promotes regeneration after damage (141)	Increase (62)	May improve dystrophic phenotype by improving repair, preventing fibrosis (142–144)

Description of what is known about effect on bone, muscle, and effect of supplementation or inhibition on DMD, if studied. All studies are preclinical animal studies unless otherwise specified.

### 2.5 Myokines

Recent studies have highlighted that myokines secreted from skeletal muscle exert regulatory effects on bone metabolism (67, 68). While the precise composition of myokines from dystrophic muscle remains to be fully elucidated, several myokines have been implicated in modulating bone turnover in DMD, including receptor activator of nuclear factor к B ligand (RANKL), fibroblast growth factor (FGF) 21, myostatin, lipocalin 2, and irisin (62). Understanding the roles of these myokines in DMD-associated osteoporosis is essential for exploring them as potential therapeutic targets. The current understanding of the roles of those myokines is summarized in **Table 1**.

#### 2.5.1 RANKL

RANKL, in conjunction with its membrane receptor RANK and the soluble decoy receptor osteoprotegerin (OPG), plays a pivotal role in osteoclast differentiation and bone remodeling (69). Notably, the expression of RANK, RANKL, and OPG is not confined to bone; all three are also expressed in muscle (70, 71). Skeletal muscle in dystrophic mouse models has been shown to exhibit elevated RANKL expression (62). While the precise function of this axis in skeletal muscle remains elusive, systemic neutralization of RANKL in dystrophic mice improved both skeletal muscle function (70) and bone strength in mdx mice (21, 72), which suggests dual function of RANKL on skeletal muscle and bone. Despite one study finding paradoxically lower levels of RANKL, OPG, and RANKL/OPG ratio in patients compared to healthy controls (73), the concept that the RANKL/RANK/OPG system may interconnect bone and muscle in DMD warrants further investigation.

#### 2.5.2 FGF21

FGF21 is a member of the endocrine FGF19 subfamily, an atypical group of FGFs secreted into systemic circulation, acting as endocrine hormones (74, 75). While FGF21 is primarily expressed by the liver and adipose tissue, it can also be secreted in large quantities from muscle tissue under pathological conditions, including mitochondrial dysfunction and muscular dystrophy (62, 76–78). In dystrophic mice, considerable amounts of FGF21 are expressed in muscle tissue and contribute to the elevated circulating levels of FGF21 (62). Blockade of FGF21 via neutralizing antibodies has shown promise in improving bone phenotype in a severe DMD mouse model (77). The impact of FGF21 on musculoskeletal system is largely unknown, with emerging evidence implicating that it may play important roles in homeostasis of both skeletal muscle and bone [as reviewed recently in (75)]. Although not fully understood, it is likely that FGF21 regulates bone homeostasis via multiple direct and indirect mechanisms. Recent studies have demonstrated that bone is a direct target of FGF21 since osteoclasts, bone marrow adipocytes, and mesenchymal stem cells express the obligate FGF21 co-receptor β-klotho (77, 79). Furthermore, FGF21 can interact with the GH/IGF-1 signaling pathway (80), which plays a crucial role in protein synthesis and bone homeostasis (81). FGF21 inhibits the action of GH/IFG-1 on proliferation and differentiation of chondrocytes directly at the growth plate (82, 83), potentially implicating its pathological role in growth retardation as well.

#### 2.5.3 Myostatin

Myostatin, also known as growth differentiation factor 8 (GDF-8), is a member of the transforming growth factor-beta (TGF-β) superfamily and is known for its inhibitory effects on both muscle and bone growth (84–86). Myostatin acts as a catabolic stimulus, resulting in muscle fiber atrophy (17). In addition to its anti-myogenic effects, it negatively regulates bone homeostasis by inducing bone resorption (85, 87) by promoting osteoclastogenesis and inhibiting osteogenesis (86, 88). Notably, the effect of myostatin on bone loss are potentiated in the context of decreased mechanical loading (87, 89, 90). Despite downregulation of myostatin in mdx mice (62) and in DMD patients (91–93), myostatin inhibition has long been considered a promising intervention to reverse pathology in muscle-wasting diseases, including DMD (94). However, there has been a failure to translate positive results of myostatin inhibition in animal models to humans (94), which will be discussed in the treatment section (Section 3).

#### 2.5.4 Irisin

Irisin, an exercise-induced myokine, is a cleaved product of secreted fibronectin type II domain containing 5 (FNDC5) (95). Unlike myostatin, irisin serves as an anabolic regulator, influencing both muscle growth and bone health (96, 97). However, the expression levels of irisin in dystrophic muscle, particularly in DMD, remains unclear. While one study reported mild decreases in serum irisin levels in Becker muscular dystrophy (98), others have found increased levels in exercised dystrophic mice (99). Another study noted reduced *Fndc5* mRNA levels in skeletal muscle of dystrophic mice, but increased protein levels, indicating intricate posttranslational regulation of FNDC5/irisin in DMD (62). The function of irisin in regulating muscle and bone homeostasis in DMD is still unknown.

#### 2.5.5 Lipocalin 2

Originally identified as an adipokine, elevated levels of lipocalin 2 have been reported in the muscles of mdx mice (100). Transgenic mice overexpressing lipocalin exhibit decreased BMD and short stature due to growth plate abnormalities (101), suggesting a potential role for lipocalin 2 in the development of osteoporosis and short stature in DMD. Inhibition of lipocalin in mdx mice via either *Lcn2* knockout or the administration of lipocalin monoclonal antibody has shown increased trabecular volume, enhanced grip strength, and decreased diaphragm fibrosis (100), indicating that it may play an important role in both skeletal muscle and bone pathologies in DMD.

#### 2.5.6 Other myokines

A plethora of other bone-regulating myokines have been identified as upregulated in dystrophic muscle. In addition to the previously mentioned myokines, leukemia inhibiting factor (LIF), secreted protein acidic and rich in cysteine (SPARC), and periostin have been found to be elevated at both the transcript and protein levels (62). However, their roles in regulating bone homeostasis in DMD or other conditions have not been studied.

Together, these findings collectively suggest that altered levels of multiple potent myokines contribute to the pathogenesis of DMD-associated bone loss. Notably, increased/decreased expression of both pro- and anti-osteogenic factors indicate a complex interplay within the skeletal muscle and bone microenvironment. Despite the complexity, the net effect of these factors is bone loss in DMD. Further research is warranted to elucidate the exact effects, underlying mechanisms, and relative contributions of these myokines, as well as to validate their clinical relevance. Understanding the interplay between these myokines and their impact on muscle function and bone health is crucial for developing targeted therapeutic interventions for DMD.

### 2.6 Vascular dysfunction

Bone is intricately connected to vascular function throughout its development, growth, maintenance, and healing processes (145). Vasculature is essential for adequate delivery of oxygen and nutrients, thus shaping the metabolic environment. Additionally, significant cross-talk exists between vascular endothelial cells and adjacent bone cells, with mutual influence on osteogenesis, bone resorption, and angiogenesis (145). Notably, impaired angiogenesis has been associated with osteoporosis (145).

Vascular dysfunction in skeletal muscle is well-documented in the context of DMD. Dystrophin, known for its structural role in cytoskeletal stabilization, binds to neuronal nitric oxide synthase (nNOS) in skeletal muscle fibers, facilitating nitric oxide (NO) synthesis for vasorelaxation (146). In DMD, the absence of dystrophin results in the loss of nNOS from the sarcolemma and its significant downregulation in the cytoplasm, leading to functional ischemia contributing to muscle damage and impaired regeneration (147). Dystrophic muscles exhibit not only vascular dysfunction, but also low vessel density and impaired angiogenesis (148–152). In addition to skeletal muscle fibers, smooth muscle cells (152–154) and endothelial cells (148, 150) also express full-length dystrophin, suggesting its crucial role in regulating vasodilation, vasocontraction, and angiogenesis [refer to recent review (155)]. However, little is known regarding the functional dysregulation of dystrophin expression out of skeletal muscle fibers in DMD. Furthermore, no studies have directly evaluated vasculature in dystrophic bone, even though it is reasonable to hypothesize that dystrophin mutations may affect bone vasculature via NOS. One study evaluating fracture healing in mdx mice observed decreased vessel density in early repair stages, correlating with delayed healing (18). The study also noted abnormally high inflammation after fracture (18), which brings to question whether impaired fracture healing was due to angiogenic dysfunction, excessive inflammation, or both. The question of whether bone exhibits vascular anomalies in DMD remains unanswered. A deeper understanding of the role of angiogenesis, vascular function, and NO^•^ signaling in DMD pathogenesis in both muscle and bone may unveil potential targets for enhancing muscular, cardiovascular, and bone health.

## 3 Treatments: current and future

Current DMD treatment is negligent to bone health. While vitamin D is often used to prevent osteoporosis, most patients still experience osteoporosis and pathological fractures (5). Bisphosphonate use is typically reserved for use following pathological fractures. Disappointing statistics regarding rates of fracture, especially those which lead to loss of ambulation, indicate that current management of bone health is inadequate in DMD patients. Furthermore, patient age is important to consider in managing bone health in DMD. In pediatric patients, growth retardation is of great concern, while in adult patients, osteoporosis and pathological fracture are of most concern. Optimal timing of therapeutic intervention requires more research.

The intricate relationship between muscle and bone along with the impact of long-term corticosteroid usage necessitate an integrated, multi-factorial approach to the management of DMD bone issues. Combining strategies to address multiple aspects of disease while minimizing off-target effects of current treatment hold the most promise. As the field gains more understanding of disease pathogenesis, more effective and targeted therapeutic avenues can be explored. Below, we will discuss potential therapeutics at varying states of investigation, which are also summarized in **Table 2**. As research continues to advance, a more comprehensive toolkit of interventions is expected to emerge.

**Table 2:** Treatments for bone comorbidities in DMD.

Conservative Treatments			
Treatment	Evidence for	Limitations	References
PT: strength training	Only exercise validated for muscle growth in other populations	Concern for exercise-induced muscle damage, ischemia, poor regeneration, negative cardiovascular effects	(13, 161–166)
PT: Aerobic exercise	Well-tolerated by DMD patients, may prolong walking	Unclear if there are any positive effects on bone	(99, 231, 232)
PT: Low-intensity vibration	Attenuates muscle and bone loss	None known	(170–172)
PT: Cyclic electrical muscle stimulation	Promising results in other immobilization conditions	Potentiated bone loss in a dystrophic mouse model	(168, 173)
PT: Assisted standing		Lacked efficacy in a small trial	(160)
Vitamin D & calcium Supplementation	Improves BMD	Unclear if vitamin D reduces fracture risk	(5, 16, 202)

Pharmacological Treatments			
Corticosteroids: Intermittent versus daily dosing	Believed to prevent adverse effects	Not as effective as daily dosing at preventing disease progression, unclear if it prevents bone loss	(31, 178–180)
*Vamorolone	No growth suppression, equal efficacy to prednisone, no effect on bone turnover markers	No direct evaluation of BMD or fracture risk in clinical trials	(182–186, 188, 233)
Bisphosphonates	Increases BMD	No strong evidence demonstrating increased fracture risk, concern for adverse effects (e.g. acute phase reaction, hypocalcemia, hypophosphatemia, adrenal insufficiency, decreased bone turnover)	(15, 34, 191, 193–197)
Denosumab	Increases BMD in patients, increases muscle strength and cardiovascular function in mice	Difficult to stop without rebound bone loss	(21, 72, 119–121, 199, 201)
GH/IGF-1 supplementation	Increases growth velocity, may be more effective in conjunction with testosterone and/or bisphosphonates	No improvement of muscle function, no effect on BMD	(203–207)
Testosterone	Preserves muscle mass, increases growth velocity, prevents bone loss, induces puberty	Benefits do not persist past termination of treatment	(212–216)
*Teriparatide/PTH	More effective than bisphosphonates at improving BMD, anabolic agent, decreases fracture risk	Not recommended before puberty	(218–220, 222)

Preclinical therapies			
*Tocilizumab (anti-IL-6)*	Prevented bone loss *in vitro*, prevents muscle degeneration in mice	Bone-protective effects have yet to be studied *in vivo*	(7, 63, 104)
*Anakinra (IL-1R antagonist)*	Attenuates bone loss in autoimmune conditions, improves forelimb strength in mice	Bone-protective effects have yet to be studied in DMD	(63, 111, 226)
*Adalimumab (anti-TNF-α)*	Attenuates bone loss in autoimmune conditions, decreases muscle degeneration in mice	May be detrimental to cardiovascular health, effect on bone has yet to be studied in DMD	(63, 107, 109, 226)
*Anti-FGF21*	Improves BMD in mice	Unknown effect on muscle, unknown adverse effects	(77)
*Anti-Lipocalin 2*	Improves bone, increases strength, decreases diaphragm degeneration	Unknown adverse effects	(100, 101)
*Irisin supplementation*	Increases muscle mass and strength, decreases degeneration in DMD models, increases bone growth and BMD in other mouse models	Unknown effect on bone in DMD	(96, 130)
*LIF supplementation*	Increases muscle repair, decreases fibrosis in DMD models, overexpression mice have increased bone deposition	Unknown adverse effects, unknown effect on bone in DMD	(142–144)
*Myostatin inhibition	Increases muscle mass, strength, and decreases fibrosis, increases bone mass and strength in mice	Not effective in clinical trials	(88, 94, 125–128, 229, 230)

Conservative treatments, pharmacological treatments, and treatments under investigation at the preclinical or clinical stage and their outcomes and limitations. *Indicates under investigation in clinical trials, italics indicates under investigation at the pre-clinical stage.

### 3.1 Monitoring bone health and early detection of fractures

Due to the high incidence of bone issues in DMD patients, frequent monitoring of bone health in this patient population is important for early detection and intervention. Vertebral fracture affects 30-60% of patients, many of which are asymptomatic (2). Current guidelines emphasize early fracture detection, and thus recommend routine lateral spine radiographs at an interval of every 1-2 years in corticosteroid-treated patients and every 2-3 years in those who are corticosteroid-naïve (2). DEXA scans may be used as an adjacent method of monitoring BMD but are not of particular emphasis since there is no established cutoff for what may warrant intervention (1, 8). Additionally, BMD Z-score may be underestimated in DMD patients since they often have a younger bone age than chronological age, which could alter estimation of fracture risk (23). Early detection of fracture, however, does not prevent fracture. Further research should be done to correlate BMD with fracture risk to establish guidelines for when to start prophylactic treatment.

### 3.2 Physical therapy

Current guidelines published by the DCCWG recommend PT as an integral part of DMD management (22). However, there is no consensus on protocols, leading to inconsistent recommendations between physicians and physical therapists based solely on provider experience and anecdotal evidence (156). Moreover, the current focus of PT is to improve or maintain muscle function and is not focused on improving bone health. Currently, only low-intensity and aerobic exercise are recommended (156–158). Investigation into modalities such as low-intensity vibration and assisted standing have been explored with some benefit but are not yet standard of care (159, 160). All of them show some benefit to maintain muscle function in clinical studies [A more detailed summary of these studies can be found in a recent review by Spaulding and Selsby (157)]. Unfortunately, no studies have evaluated any type of exercise for improving bone health in DMD patients (156).

When discussing PT to improve bone health in healthy populations, high-load resistance training or repetitive high-impact movements are optimal to increase muscle mass and increase BMD (13). However, for DMD patients, there are several concerns, including exercise-induced muscle damage, ischemia, regenerative ability of muscle, and effects on cardiovascular function. It is recommended to provide sub-maximal loading to reduce the risk of muscle damage (156). However, the scientific basis of this recommendation is not definitive. Numerous studies have demonstrated that dystrophic muscle is more prone to damage following exercise than healthy muscle (161–163), and satellite cells in dystrophic muscle have impaired regenerative capacity (164–166). However, some studies also indicate that dystrophic muscle has a high functional regenerative capacity and recovers function well after contractile injury (162, 167), indicating that higher intense exercise may not lead to a functional deficiency as we thought. There is certainly more to investigate regarding exercise regimens and regenerative capacity of dystrophic muscle and how that may affect bone.

In addition to exercise, some modalities, such as low-intensity vibration, assisted standing, repetitive electrical muscle stimulation, and high-dose compressive loading, have shown benefits in other muscle-wasting and immobility related bone conditions (168, 169). Of these, electrical muscle stimulation and low-intensity vibration can also attenuate muscle loss (170–172), thus potentially addressing both muscle and bone problems in DMD. So far, electrical muscle stimulation has been evaluated in mice, while low-intensity vibration and assisted standing have been evaluated in humans. One study found that electrical muscle stimulation, which provides cyclic loading, worsened bone loss in mdx mice, potentially by increasing release of harmful myokines (173). This has not been evaluated in humans with DMD, so it is unclear if this result would hold. In patients, assisted standing, meant to return body-weight stimulus to bone, did not prevent bone loss in four wheelchair-bound DMD patients (160). Low-intensity vibration is FDA-approved to treat osteoporosis in adults and is thought to increase BMD by stimulating osteocytes and mesenchymal stem cells, although the exact mechanism is unclear (170, 171). It has also shown some efficacy in increasing BMD in DMD patients and in other pediatric populations and is generally well-tolerated (13, 159, 174), warranting further exploration of this modality.

The optimal PT protocols to promote maintenance or gain of muscle function and bone still require much more mechanistic and clinical exploration. The complexities of muscle damage and regeneration, its crosstalk with bone, differences in the response of muscle and bone to different types of exercise, and potential confounders of vascular dysfunction, leave many unanswered questions. The optimal protocol may not be intuitive, and likely should include additional modalities targeting bone.

### 3.3 Optimal corticosteroid treatment and alternatives

Corticosteroids play a pivotal role in preserving muscle function by mitigating inflammation; however, their usage is accompanied by a range of adverse effects, such as excessive weight gain, adrenal insufficiency, behavioral changes, Cushing’s syndrome, stunted growth, and osteoporosis. The optimization of corticosteroid administration including the choice of agent, dosing, timing, and frequency remains a dynamic area of research in the field, as recently reviewed by Biggar et al. (175).

Intermittent dosing of corticosteroids has been explored as an alternative to daily administration with hopes of mitigating side effects while maintaining efficacy. However, results have been inconsistent. Studies have found that intermittent dosing is at least better than placebo (176, 177), but it is not as effective at slowing disease progression as daily dosing (31, 178–180). One RCT of weekend versus daily dosing found a statistically significant increase in growth and BMD in the weekend group compared to the daily group, but patients’ heights and BMD were not statistically significantly different at the end of the study (179). Despite this being a theoretically appealing option, evidence suggests that intermittent dosing is inferior for disease progression and does not substantially reduce the risk of bone loss.

Ongoing efforts in treatment development focus on generating more selective corticosteroids to balance efficacy and side effects. Vamorolone is a synthetic corticosteroid which is more selective for the glucocorticoid receptor while antagonizing the mineralocorticoid receptor, which should theoretically reduce side effects, especially bone-related side effects (181). In preclinical studies, vamorolone improved skeletal muscle repair with minimal impact on hormonal regulation, growth, and immune suppression (181–183). Phase III clinical trials have shown comparable or superior efficacy to prednisone in retaining muscle function while preventing growth suppression (184–186). Vamorolone’s potential bone-sparing properties are also noteworthy, as evidenced by its neglectable effects on bone growth and trabecular thickness in mice (181, 187) and decreased markers of bone turnover in patients (186, 188). However, further clinical investigations are required to ascertain whether it is truly less detrimental to bone than prednisone.

### 3.4 Anti-resorptive therapy

Anti-resorptive therapies, such as bisphosphonates and denosumab, are widely used for osteoporosis treatment (189, 190). There is currently no consensus on the use of antiresorptive therapies to manage osteoporosis in DMD, but bisphosphonates are commonly used following severe fracture (2, 191). Bisphosphonates inhibit osteoclast attachment to the bone surface and induce cell apoptosis (190), while denosumab, a monoclonal antibody targeting RANKL, prevents osteoclast formation (192).

DCCWG recommends intravenous bisphosphonate treatment following a pathological fracture (vertebral Genant grade 2 or 3 or low-trauma long bone fracture) (2). However, it is still unclear whether bisphosphonates are beneficial as a prophylactic treatment. Landfeldt et al. recently published a systematic review on bisphosphonate use in corticosteroid-treated DMD patients (191). Notably, there is good quality evidence that bisphosphonate use increases BMD in DMD patients, but it is unclear if this translates to a lower fracture risk (15, 34, 191, 193, 194). Unfortunately, the rate of adverse effects in DMD patients is high, with acute phase reaction, hypocalcemia and hypophosphatemia, and precipitation of adrenal insufficiency of particular concern (191, 195). Of further concern, histomorphometric analyses indicate that bisphosphonates globally decrease bone turnover (1, 196, 197), which is problematic during bone development (198). Currently, bisphosphonates are one of the few options for treating osteoporosis but may not be the best option for young DMD patients.

Denosumab has also been used in pediatric populations (189) and has shown promising results in DMD. Case reports demonstrate improved BMD without notable side effects, indicating its relative effectiveness and tolerability in children (120, 121, 189). Notably, denosumab and other anti-RANKL therapies may also improve muscle strength and cardiac function, as reported in mouse models of DMD (21, 72, 119, 199). One consideration when starting denosumab treatment is that it cannot be stopped abruptly; its effects on BMD decline rapidly following termination of treatment (72, 200, 201). This is easily remedied by a short course of bisphosphonates (72, 200, 201). Given the favorable outcomes observed in both muscle and bone in DMD patients and animal models, further investigation into the use of denosumab for this population is warranted.

### 3.4 Vitamin D and calcium supplementation

Calcium and vitamin D supplementation are strongly recommended for DMD patients (22). DMD patients are particularly susceptible to vitamin D deficiency because of corticosteroid use, obesity, and low levels of vitamin D binding protein, meaning supplementation needs to be aggressive (2000 IU/day) to reach adequate levels in most patients (53). Cohort studies do not find an association between vitamin D supplementation and fracture risk, but an RCT found that adequate supplementation improved BMD (5, 16, 202). Although vitamin D supplementation alone does not fully prevent osteoporosis in DMD (5), due to its simplicity, low risk, and possible benefit, vitamin D supplementation should be a pillar of maintaining bone health in DMD patients.

### 3.5 Hormone therapy

Endocrine management is an important aspect of DMD treatment given the hormonal imbalances induced by corticosteroids. GH and testosterone therapy have been investigated to restore hormonal levels to normal ranges and promote linear growth. Additionally, to improve bone health, PTH therapy via teriparatide, a PTH analogue, has been explored as a strategy specifically to improve bone quality and prevent osteoporosis.

The role of GH in bone problems in DMD has been extensively studied, particularly due to growth retardation observed in DMD patients. Although there is no clear evidence of GH dysregulation in DMD patients who are not treated with corticosteroids (44), corticosteroid use is well-known to impair the GH axis and GH secretion (42, 51). Both GH supplementation and inhibition have been tested in DMD patients, but yielded controversial and inconclusive results. Supplementation, either via recombinant human GH (rhGH) or IGF-1, improved in growth velocity but failed to demonstrate motor function improvement (203–205). Moreover, there has been no documented improvement in BMD with GH or IGF-1 treatment in mdx mice or patients, possibly due to the short duration of these trials. GH secretagogues have been explored in mice, including ghrelin and the synthetic secretagogues EP80317 and JMV2894. Both studies demonstrated decreased muscle fibrosis and inflammation, and increased muscle strength (206, 207), but neither study evaluated the bone phenotype in these mice. Conversely, the idea that GH inhibition might be beneficial stemmed from the observation that a patient with DMD and GH deficiency had no clinical evidence of muscular weakness before initiation of GH replacement therapy; and treatment with human GH resulted in appearance of symptoms of easy fatigability and proximal muscle weakness, which suggest that increased growth may worsen muscle function in DMD (208). However, clinical trials investigating mazindol, a postulated inhibitor of growth hormone release, have shown inconsistent effects on growth suppression and no benefits on muscle strength (209, 210). In light of limited data and ongoing controversy, routine use of rhGH or secretagogues in DMD population is not recommended. Any decision to use rhGH should be made on a case-by-case assessment with biochemical evidence of growth hormone deficiency, weighing the risks and benefits.

Delayed or absent pubertal development in DMD patients on glucocorticoids is common due to hypogonadotropic hypogonadism (211). Testosterone therapy is recommended for pubertal induction in these individuals, typically initiated by age 14 (22, 212–214). Clinical trials have shown that testosterone therapy not only induces puberty but also preserves muscle mass, improves growth velocity, and prevents bone deterioration (212–216). However, these benefits are observed only during treatment. A recent follow up study on patients that were treated with testosterone for 2 years demonstrated persistent lower testosterone levels and testicular volumes than adult reference values; muscle functional volume increased during the intervention but declined in the years after cessation of supplementation (214). Given the known additional advantages of testosterone on bone health, muscle, and well-being (217), the benefits of restoring testosterone to normal physiologic levels are deemed to outweigh potential risks. Nevertheless, further research is warranted to elucidate the optimal timing, duration, and regimen for testosterone supplementation in DMD patients and its potential impact on bone health.

PTH administration in DMD mouse models improved or maintained BMD in several studies (218–220). Research on corticosteroid-induced bone loss suggests that PTH may be more effective than bisphosphonates (221). Although its safety and efficacy in improving bone health in DMD still need to be validated in clinical trials, a small cohort treated with teriparatide, a PTH analogue, demonstrated decreased fracture risk (218, 222).

As previously mentioned, multiple hormonal pathways are affected in DMD. This complexity prompted an investigation to evaluate the efficacy of hGH, testosterone, and zolendronic acid (ZA, a bisphosphonate) in various combinations after vertebral fractures (15). The study revealed that hGH and testosterone, either alone or in conjunction with ZA, significantly delayed the occurrence of subsequent vertebral fractures compared to ZA treatment alone. Moreover, multivariate analysis demonstrated that hGH was the greatest contributor in enhancing this protective effect (15). This study highlights the intricate hormonal imbalances contributing to osteoporosis in DMD patients, which point out the need for a holistic management strategy that targets these hormonal disruptions.

### 3.6 Novel anti-inflammatory and immunomodulatory agents

In addition to corticosteroids, monoclonal antibodies targeting specific immune modulators are increasingly used to treat inflammatory conditions. Tocilizumab, an anti-IL-6 receptor monoclonal antibody, is used to treat certain autoimmune conditions and may attenuate bone loss (223–225). *Ex vivo* and *in vitro* experiments found that tocilizumab rescued the anti-osteogenic/pro-osteoclastogenic effect induced by DMD patient serum (7). In mdx mice, tocilizumab treatment improved skeletal muscle phenotype by improving muscle diameter, reducing fibrosis, and decreasing serum CK, suggesting it has promise for altering disease progression (104). Although no studies have evaluated its effect on bone *in vivo*, this may be a promising avenue to treat muscle and bone degeneration. Anti-IL-1 receptor antagonist anakinra and anti-TNF-α monoclonal antibody adalimumab are also used to treat autoimmune diseases and may also attenuate bone loss (63, 226). Neither has been tested for effects on bone in DMD models, but they have been studied for their effects on muscle. Anti-TNF-α treatment decreased muscle damage, degeneration, and fibrosis in mdx mice, but appeared detrimental to cardiac function (107, 109). Anti-IL-1R treatment in mdx mice improved forelimb strength (111). The effects of these treatments on disease progression and osteoporosis in DMD requires more investigation, but thus far tocilizumab appears to be most promising.

### 3.7 Novel therapies targeting myokines

Myokines secreted from dystrophic muscle significantly contribute to the development of bone abnormalities in DMD. Targeting these myokines holds considerable promise for mitigating dystrophic bone loss. Of the myokines discussed, RANKL, FGF21, myostatin, irisin, and lipocalin 2 have been studied in mouse models. Only myostatin inhibition has been translated to the clinic.

In preclinical studies, inhibition of FGF21 or lipocalin 2, and supplementation of irisin or LIF have been studied with some promising results. Inhibition of FGF21 via neutralizing antibody increased BMD in a severe DMD mouse model by decreasing osteoclastogenesis (77). The effect of FGF21 inhibition on dystrophic muscle has yet to be determined. Lipocalin 2 has been studied in dystrophic mice by neutralizing antibody and global knockout of *lcn2*, which resulted in increased trabecular volume, grip strength, and reduced diaphragm fibrosis (100). A study by *Reza* et al. (130) revealed that recombinant irisin treatment increased muscle mass and strength in addition to reducing muscle fibrosis and necrosis, although its effects on bone are unknown. Several studies have also evaluated LIF supplementation, which improved dystrophic phenotype by improving repair and preventing fibrosis (142–144), but given its role in promoting solid tumor progression, further research should be cautious of potential oncogenic effects (227). With the popularity of monoclonal antibody treatments, inhibition of FGF21 and lipocalin 2 could be promising therapeutic targets. Irisin could be supplemented by recombinant protein or mimetics (97, 228). While the effects of FGF21 on muscle and irisin and LIF on bone are unstudied, their known roles in muscle and bone pathology warrant further investigation in pre-clinical models.

In preclinical studies involving DMD animal models, myostatin inhibition led to increased muscle mass and strength, and decreased fibrosis (125–128, 229), as well as improved bone mass and strength (88). However, these positive results have not translated to positive outcomes in clinical trials (230). Three distinct myostatin inhibitors have been developed and all have failed in clinical trials [as recently reviewed in (94, 230)]. The discrepancy of efficacy between animal models and humans is likely due to the differing expression levels of myostatin between mdx mice and human patients. Myostatin levels in mice are approximately 5 to 9-fold higher than in humans (124). Thus, further exploring direct inhibition of myostatin is unlikely to be fruitful. However, increased understanding of myostatin action, such as downstream signaling pathways, may provide alternative druggable targets.

## 4 Conclusions

The pathogenesis of bone abnormalities in DMD is multifactorial, extending beyond corticosteroid usage and loss of ambulation. While factors such as mechanical loading, corticosteroid usage, nutritional deficiencies, hormonal imbalances, systemic inflammation, and myokine dysregulation contribute to bone health deterioration in DMD patients, the precise interplay among these factors requires further elucidation. Moreover, the management of bone health in DMD necessitates a multidisciplinary approach that includes PT, nutritional supplementation, hormonal therapy, anti-inflammatory interventions, and potential myokine-targeted therapies. It is evident that a more comprehensive clinical management plan is imperative to address the complex pathophysiology of bone abnormalities in DMD and must take into account patient age. Future research efforts should focus on unraveling the underlying mechanisms driving bone pathology in DMD and developing tailored therapeutic strategies to mitigate bone loss and improve skeletal health outcomes.
